# Supplementary material for: Linking photoelectron circular dichroism to the asymmetric total photoemission yield measured in aerosol nanoparticles of tyrosine
Source: Nat Commun. 2026 Mar 24;17:2792. doi: 10.1038/s41467-026-70997-w (PMC13018607; doi:10.1038/s41467-026-70997-w)
Supplement: Supplementary file 1 — Supplementary Information [file 41467_2026_70997_MOESM1_ESM.pdf]

Supplementary Information for

Linking photoelectron circular dichroism to the asymmetric total photoemission yield measured in aerosol nanoparticles of tyrosine

Sebastian Hartweg, Dušan K. Božanić, Gustavo A. Garcia and Laurent Nahon

Supplementary Note 1. Electron microscopy images of tyrosine aerosol particles

Supplementary figure 1 shows typical scanning electron microscopy images of tyrosine aerosol particles deposited on a conductive substrate. Particles of needle-like morphology can be observed in a broad range of particle sizes, which are typical for tyrosine samples of both synthetic and biological origin. These structures suggest formation of orthorhombic tyrosine crystals in the deposited aerosol sample. The images clearly show some large particles exceeding 1 μm in diameter that are formed by coagulation of particles on the substrate. These particles, however, are not transmitted by the aerodynamics lens system, and, therefore, do not contribute to our experimental photoelectron images. Smaller, spherical, and indicatively amorphous particles were also observed suggesting semi crystalline nature of the sample. The formed aerosol seem to differ from the presumably more crystalline particles studied by Paul and Siegmann[41], which was likely caused by slower formation process applied by these authors.

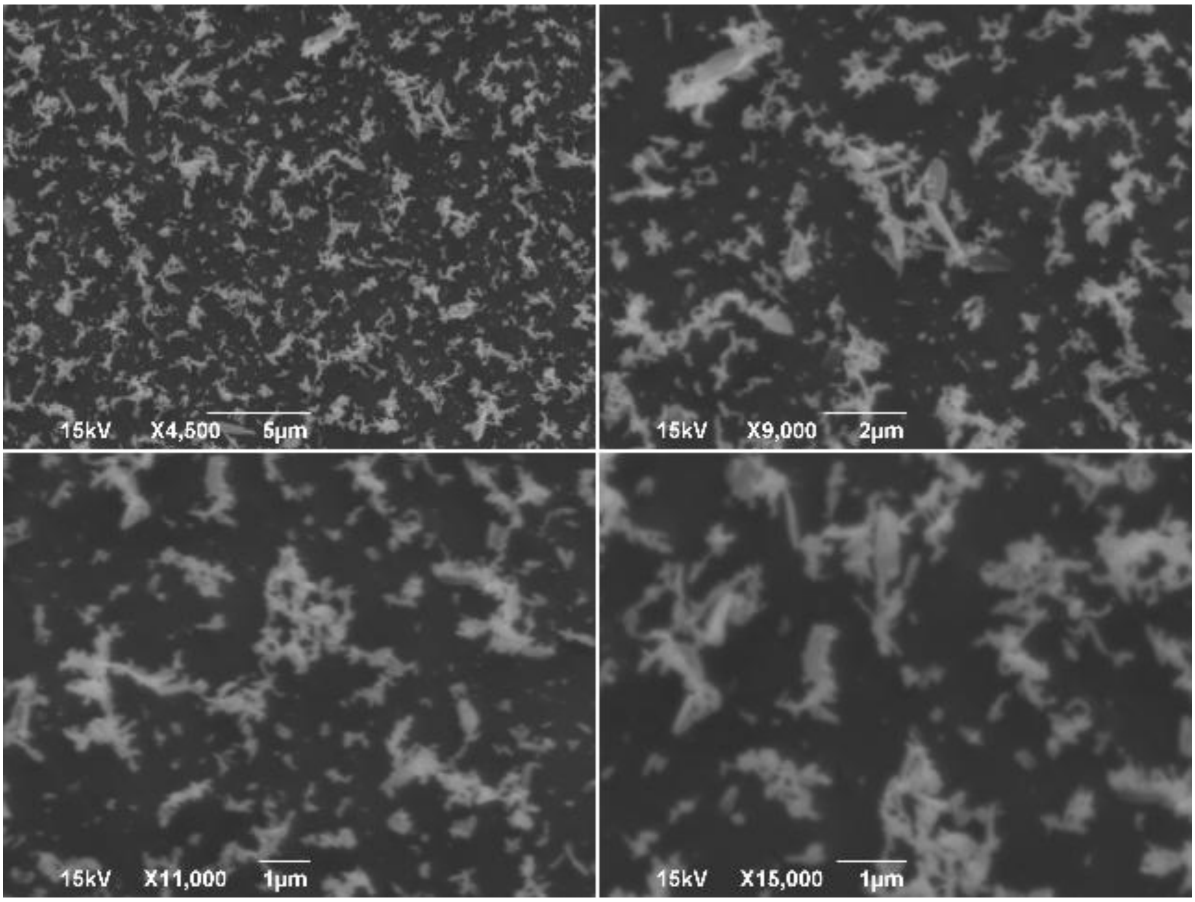

Supplementary figure 1. Morphology of tyrosine aerosol particles.
Scanning electron microscopy images of deposited tyrosine aerosol particles.

Supplementary Note 2. Discussion of effects of light intensity and crystallinity of particles on expected chiral asymmetry of photoemission yield

While our simulation reproduces the particle size dependence of the effect observed by Paul and Siegmann[41], their study also reported an increasing asymmetry for low laser intensities and a higher degree of crystallinity of the particles. Based on the comparison of the electron microscopy images recorded of our particles (Supplementary Figure S1) with those reported by Paul and Siegmann, the particles in our study are assumed to be predominantly amorphous or more polycrystalline in nature. A higher degree of crystallinity can affect light absorption properties, as well as the size and the morphology of the aerosol particles. These aspects can lead to differences in the resulting light intensity distributions within the particles and therefore affect the shadowing effect and the obtained CAPY. More importantly, the PECD effect is known to be highly sensitive to molecular conformation, as demonstrated on several isolated chiral species[6, 7, 10, 28, 65]. As such, the formation of an ordered crystal lattice, possibly affecting the number of populated molecular conformers and creating a structured network can affect the magnitude of the PECD. In addition, if polycrystalline Tyr contributions are present they might enhance the PECD effect because of a possible supramolecular chirality effect.

The decrease of the observed CAPY for high light intensities was attributed by Paul and Siegmann to multiple ionization of single nanoparticles. Emission of electrons from already ionized particles leads to the retention of the slowest photoelectrons in the created Coulomb potential. Alternatively, multiphoton absorption can lead to the ejection of photoelectrons from lower-lying molecular orbitals, that may show a lower PECD, thus reducing the chiral asymmetry also in the photoemission yield. In conclusion, the effects of chiral asymmetry of the photoionization yield due to PECD and nanoparticle shadowing can explain all experimental observations made previously. Most importantly, the fact that PECD is contained in the electric dipole approximation of light-matter interaction explains the impressive magnitude of the effect, that would be truly unusual for an effect requiring magnetic and electric dipole contributions (E1M1) such as conventional CD.

Supplementary Note 3. Comparison between the simulated and theoretical CAPY data.

Supplementary figure 2. illustrates the g^{E1} (α) dependances for $|b_1^p|$=0.0125, 0.025, and 0.05 reached by simulation (Figure 4 and Figure 5 in the main text) and by analytical formula (Eq. (6)) obtained by integration of the assumed photoelectron angular distribution (PAD) for chiral aerosol particles. (Eq. (2)). Within the error margin, which arises from finite variable sampling in the simulations and the chosen value of the electron escape length in the simulation, the results produced by both methods align closely. This further confirms demonstrates the validity of presumed PAD accurately describes the chiral asymmetry observed in photoemission from aerosol nanoparticles.

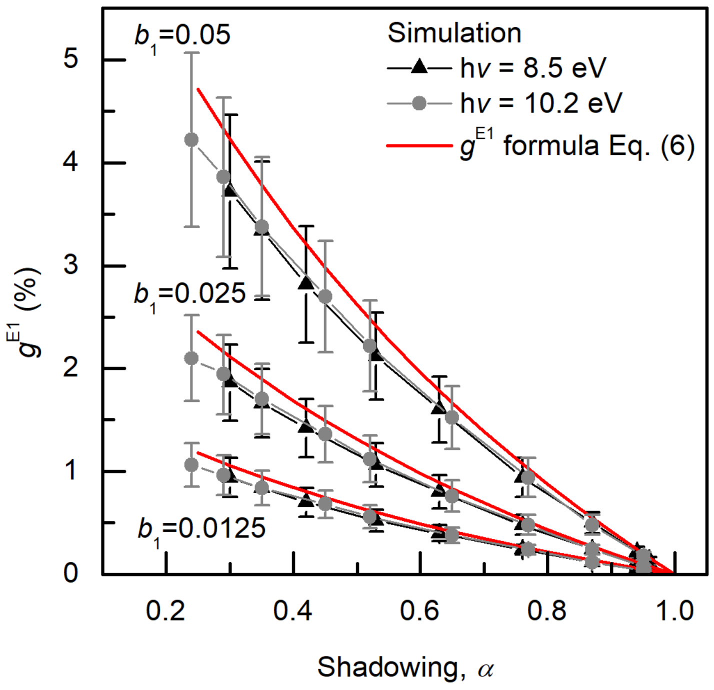

Supplementary figure 2. Comparison between the two theoretical models.
Comparison between the g^{E1} values obtained by simulation (scatter plots with error bars) and by analytical formula (red lines, Eq. (6) in the main text) for b_1 of 0.0125, 0.025, and 0.05. The error bars of g^{E1} were estimated to ≈20% of the values based on the uncertainties due to finite variable sampling in the simulations and the electron escape length value used.
